# Lassa virus diversity and feasibility for universal prophylactic vaccine

**DOI:** 10.12688/f1000research.16989.1

**Published:** 2019-01-31

**Authors:** Igor S. Lukashevich, Slobodan Paessler, Juan Carlos de la Torre

**Affiliations:** 1 Department of Pharmacology and Toxicology, University of Louisville, Louisville, KY, 40292, USA; 2 Department of Pathology, University of Texas Medical Branch at Galveston, Galveston, TX, 77555, USA; 3 Department of Immunology and Microbiology IMM-6, The Scripps Research Institute, La Jolla, CA, 92037, USA

**Keywords:** Lassa virus, Lassa fever, vaccine development

## Abstract

Lassa virus (LASV) is a highly prevalent mammarenavirus in West Africa and is maintained in nature in a persistently infected rodent host, *Mastomys natalensis*, which is widely spread in sub-Saharan Africa. LASV infection of humans can cause Lassa fever (LF), a disease associated with high morbidity and significant mortality. Recent evidence indicates an LASV expansion outside its traditional endemic areas. In 2017, the World Health Organization (WHO) included LASV in top-priority pathogens and released a Target Product Profile (TPP) for vaccine development. Likewise, in 2018, the US Food and Drug Administration added LF to a priority review voucher program to encourage the development of preventive and therapeutics measures. In this article, we review recent progress in LASV vaccine research and development with a focus on the impact of LASV genetic and biological diversity on the design and development of vaccine candidates meeting the WHO’s TPP for an LASV vaccine.

## Introduction

Lassa virus (LASV) was originally isolated from a nurse (Lily Pinneo; strain LP) in 1969 during a nosocomial outbreak in a mission hospital in the city of Jos in north-central Nigeria ^1, 2^. Electron microscopy of LASV-infected Vero cells revealed morphological similarities with the prototypic arenavirus lymphocytic choriomeningitis virus (LCMV) ^3, 4^. Currently, LASV belongs to the Old World group (OW or LCMV-LASV serocomplex) of the genus *Mammarenavirus* in the *Arenaviridae* family ^3^.

Persistently infected *Mastomys natalensis* is the main source of natural LASV infection of humans ^5, 6^. Rodent-to-human transmission occurs by the inhalation of dust contaminated by the excreta of infected rodents, direct contact of abraded skin with contaminated material, or the ingestion of contaminated food ^7^. Clusters of infected *M*. *natalensis* are responsible for spatial distribution of Lassa fever (LF) cases in endemic areas ^8– 11^. LASV infection is confined predominantly to *M. natalensis* monophylogenic group A-I in West Africa ^12^, whereas other phylogroups from Central and Southern African regions have been associated with the non-pathogenic OW arenaviruses Mopeia (MOPV), Morogoro (MORV), Gairo (GAIV), and Luna (LUNV) ^13– 16^. The detection of LASV in eastern Nigeria in *M. natalensis* from phylogroup A-II, which occupies a territory extending up to eastern Congo, underlines the potential of LASV to infect other genetically related host species and expand its geographic distribution ^12, 17, 18^. In addition, the isolation of LASV from *Mastomys erythroleucus* and *Hylomyscus pamfi* in Guinea and Nigeria, respectively, suggests that host-switching events potentially contribute to LASV geographic expansion ^17, 19– 21^. LASV-endemic areas cover about 80% of Sierra Leone (SL) and Liberia, 50% of Guinea, 40% of Nigeria, 30% of each of Ivory Coast, Togo, and Benin, and 10% of Ghana with an at-risk population as high as 200 million people ^20, 22, 23^. Moreover, West Africa is undergoing rapid demographic and environmental changes that are likely to increase LASV spillover events in coming decades ^10^. The estimated global burden of LF is the highest among viral hemorrhagic fevers with the exception of dengue fever ^24^.

Most LASV human infections are asymptomatic or cause mild flu-like illness, but about 20% can result in manifested illness, which can progress to severe multi-organ failure, hypovolemic sepsis-like shock, and death. The overall estimated mortality rate for “rural” LASV infections is 1% to 2% ^25– 28^. Among hospitalized patients and some vulnerable groups (women in late pregnancies, children under 5 years, and individuals with immune deficiencies), LF fatality can be 50% or higher.

LASV infection has been largely ignored as a public health threat ^11, 29^. However, during 2015–2016, historically high (59.6%) mortality among laboratory-confirmed LF cases was reported in Nigeria ^30, 31^, triggering the re-evaluation of LF risk for global health security ^11, 32^. In 2017, the World Health Organization (WHO) identified LASV as a top-priority pathogen for fast-track research and vaccine development ^33^. From January through the middle of April 2018, 1,849 suspected LF cases were reported across 21 states of Nigeria with 25.4% fatality among confirmed cases ^34^, prompting the WHO to declare a public health emergency ^35^.

## Lassa virus genetic diversity

LASV has a bi-segmented single-strand negative-sense RNA genome ^36^. The large (L) RNA encodes for the L protein, an RNA-dependent RNA polymerase, and for the matrix Z protein. The small (S) RNA encodes for the nucleoprotein (NP) and enveloped glycoprotein precursor (GPC), which is processed in infected cells into stable signal peptide (SSP) and the mature GP1 (attachment) and GP2 (fusion) glycoproteins.

Molecular dating indicates that LASV originated in Nigeria about 1,000 years ago and gradually moved westwards ^6, 37– 39^. Initial phylogenetic analysis revealed that LASV sequences clustered geographically independently of a rodent or human source and formed four major phylogenetic lineages ^40^. Lineages I–III comprise LASV strains isolated in different geographic areas of Nigeria. The largest lineage, lineage IV, with the prototypic JOSIAH/SL/76/H (JOS) includes strains from Guinea, Liberia, and SL. Genetic analysis of clinical LF samples collected in 2008–2013 confirmed the existence of four major LASV lineages and provided additional evidence for high LASV genetic diversity, up to 25% and 32% for the S and L RNA segments, respectively ^38, 41^. A lineage V, which has a sister relationship with lineage IV, has been proposed for LASV isolates from Mali and Ivory Coast ^39, 42^. In addition, a Togo isolate with a mosaic genome structure (the L segment related to clade II and the S segment related to lineages I and IV) has been proposed to represent a separate lineage VI ^43^. No evidence for LASV recombination has been presented so far, and naturally occurring LASV reassortment seems to be a rare event, since only three reassortants were identified among 194 LASV RNA clinical samples ^38^. LASV geography-based clustering suggests multiple infections of individuals travelling across different LASV-endemic areas as the most likely explanation for the observed LASV reassortants ^6^.

## Lassa virus genetic diversity and differences in clinical manifestations of Lassa fever disease

Most LASV animal studies have been carried out with JOS or JOS-related strains from the same lineage (IV). Accordingly, there is very limited knowledge about the pathogenicity of Nigerian strains in guinea pigs and non-human primates (NHPs), which poses an obstacle for LASV vaccine development ^44^. The LASV/JOS (lineage IV) and LASV/803213/74/H (lineage II) are highly pathogenic for strain 13 guinea pigs ^45– 49^. However, the prototypic LASV/LP (lineage I) does not cause fatal disease in these animals ^48^. LASV/GA391/NIG/77/H (lineage III) is lethal for outbred guinea pigs ^50^, whereas wild-type LASV/JOS kills only 30% to 40% of outbred guinea pigs ^49^. Notably, there is a poor correlation between the clinical outcome of LF in humans and the virulence of LASV in guinea pigs ^51^.

**Figure 1.**
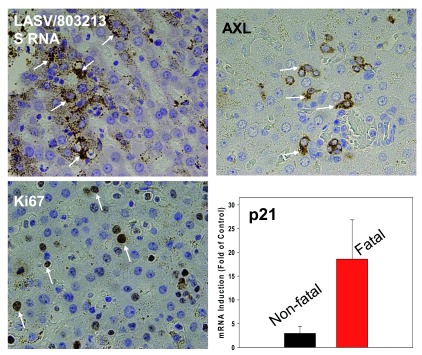
Nigerian strain of LASV induces LF-like human hepatitis with biomarkers of pathological hepatocyte proliferation. Infection of common marmosets with LASV/803213 (lineage II) induced fatal hepatitis clinically and histologically similar to hepatitis caused by LASV/JOS (lineage IV) ^66^. ( **A**) LASV S RNA of strain 803213 was detected by using RNAscope *in situ* hybridization and amplification (Advanced Cell Diagnostics, Inc.). Positively stained brown spots in liver tissues of LASV-infected marmosets (but not in tissues of mock-infected animals, not shown) are indicated by arrows. ( **B**, **C**) Staining for AXL (additional LASV cell receptor) and Ki67 (hepatocyte proliferation marker) was performed as previously described ^57^. Positively stained cells are indicated by arrows. Tissues of mock-infected marmosets were negatively stained on these markers (not shown). ( **D**) Detection of cell cycling p21 gene expression in fatally infected marmosets versus non-fatal (vaccinated) survivors was measured by quantitative reverse transcription polymerase chain reaction.

LASV/JOS is highly pathogenic in NHPs and reproduces major biological markers of fatal human LF cases ^52– 54^. However, a recent LASV/JOS-related isolate from a case of LF in Mali was less pathogenic in cynomolgus macaques than LASV strains from SL and Liberia ^55^. LASV/803213 (lineage II) is the only Nigerian strain tested in NHPs and caused fatal disease with predominant liver involvement in line with a recent clinical report ^56^ and experimental arenavirus-induced liver pathology characterized by aborted hepatocyte proliferation and upregulation of Axl-1 ^57^, an additional LASV receptor ( Figure 1).

Clinical studies in SL and Liberia during the 1970s and 1980s ^1, 58– 60^ established fever, pharyngitis, retrosternal pain, and proteinuria as the best predictors of LF, whereas the best predictor of outcome was fever, sore throat, and vomiting. Elevated levels of aspartate aminotransferase (AST) in plasma and high viremia were strongly associated with death ^25^. A high aspartate AST/alanine aminotransferase (AST/ALT) ratio suggested substantial involvement of non-hepatic tissues in LF pathogenesis. Low or undetectable levels of pro-inflammatory chemokines interleukin-8 (IL-8) and interferon (IFN)-inducible protein 10 (IP-10) and elevated levels of tumor necrosis factor-alpha (TNF-α) receptors and IL-6 were associated with fatal LF ^61^. High IL-6 in plasma is considered a biological marker of fatal LF linked with liver pathological regeneration ^52, 62, 63^. Likewise, low levels of IL-6, IL-10, CD40L, AST, ALT, alkaline phosphatase, and blood urea nitrogen in LF patients from SL correlated with survival. At the terminal stage, LF is characterized by severe pulmonary edema and acute respiratory distress, and signs of encephalopathy are observed with coma and seizures ^28^.

A retrospective study on a small cohort of patients with LF treated during 2012–2013 in Jos hospital, north-central Nigeria, confirmed fever, hemorrhagic manifestations, cough, proteinuria, and retrosternal pain as clinical predictors of LF ^64^. During the 2016 transmission season, patients with LF were predominantly from urban areas and fever was not a leading clinical sign, whereas bleeding diathesis, abdominal pain, and headache were present in more than 50% of confirmed cases ^65^. Recent studies on a larger cohort of patients with LF treated in Irrua Specialist Teaching Hospital (Edo State, Nigeria) ^56^ revealed similarities and differences between LF in SL and Nigeria. Both groups of patients had liver pathology manifestations, but Nigerian patients often had a lower AST/ALT ratio consistent with hepatocyte involvement. In both groups, higher viral load strongly correlated with poor outcome ^25, 65^. However, in contrast to SL LF, sepsis-associated hypothermia rather than fever was a predictor of poor outcome in Nigerian patients ^67^. LF patients younger than 5 or older than 55 were the most likely to die ^30, 56^. Kidney pathology, manifested as high creatinine and urea blood levels, was one of the most notable clinical features of Nigerian LF ^56^. Analysis of 2017–2018 Nigerian LASV strains did not reveal differences in LASV genetic lineages or the epidemiology of the disease compared with previous years ^68, 69^. The reason for the recent LF increase in Nigeria is not clear but is potentially related to changes in rodent reservoir or improvements in diagnostics and public awareness or both.

Sensorineural hearing loss (SNHL) seems to be a common feature of LASV infection in West Africa, and up to 80% of individuals who experienced deafness had LASV-specific antibodies versus 19% of matched controls ^22, 70, 71^. In a murine model of SNHL, LASV isolates from fatal or non-lethal human cases affected cochlear hair cells and induced degeneration of the spiral ganglion cells of the auditory nerve ^71, 72^. This model will provide valuable insights into the mechanisms of SNHL in humans. In NHP survivors, a recent study documented the presence of severe vascular lesions in inner ear tissues ^73^.

## Immunity to Lassa virus and correlates of protection against Lassa fever

Studies involving LF survivors and validated animal models of LASV infection indicate that early and robust virus-specific CD4 ^+^ and CD8 ^+^ T-cell responses are the best correlates of protection but that neutralizing antibodies appear too late after infection and at low titers ^52, 54, 74– 78^. Moreover, individuals who experienced subclinical or mild forms of LF are susceptible to re-infection with LASV but have long-term protection against LF disease ^7, 75, 79, 80^. Notably, LF survivors from Guinea had strong memory CD4 ^+^ T-cell responses against conserved epitopes in NP and GP2 from LASV/JOS and Nigerian LASV strains ^81, 82^. Nevertheless, potent LASV neutralizing monoclonal antibodies can be isolated from LF survivors, and these antibodies were protective in animal models of LF ^83, 84^. Consequently, a replication-competent and deeply attenuated vaccine capable of inducing the right combination of both cellular and humoral responses would be the preferred candidate.

## Feasibility of a pan-Lassa virus vaccine

LASV genetic diversity is a great challenge for the design of a “universal” preventive LF vaccine. Only one of three predicted cross-reactive HLA-A2-restricted LASV-GPC CD8 ^+^ T-cell epitopes is shared between LASV/JOS (lineage IV) and LASV/GA391 (lineage III), and CD8 ^+^ T cells from JOS-immunized transgenic mice did not recognize GA391-derived GPC epitopes ^85^. However, studies with the vaccine candidate reassortant ML29, carrying the L RNA from MOPV and the S RNA from LASV/JOS encoding the major protective antigens NP and GPC ^86^, provided the first evidence supporting the feasibility of a pan-LASV vaccine. The expression of NP is an important feature of ML29 contributing to cross-protection ^46, 87, 88^, which is consistent with the observation that NP-specific CD8 ^+^ T cells play a major role in virus control in mice infected with LCMV ^89^, and LF survivors had strong CD4 ^+^ T-cell responses recognizing conserved and variable epitopes of the LASV NP ^81^. These findings suggest that anti-NP response at an early stage effectively controls infection and contributes to cross-protective immunity.

A single immunization with ML29 fully protected guinea pigs and marmosets against fatal disease caused by LASV/JOS (lineage IV) and Nigerian strain 803213 (lineage II) ^46, 88^. In Rhesus macaques, on day 28 after ML29 immunization, up to 13% of spleen cells secreted IFN-γ after stimulation with distantly related LCMV ^87^. LASV shares about 50% sequence homology with LCMV, including conserved NP and GPC T-cell epitopes ^90^. However, naïve macaques challenged with the WE strain of LCMV died from an LF-like disease, whereas all ML29-vaccinated primates, including simian immunodeficiency virus (SIV)-infected monkeys, were protected against fatal LF-like disease ^88, 91– 93^. These results indicate that ML29 is safe and immunogenic and induces broad T-cell-mediated cross-protective immunity. Among available LASV vaccine candidates, safety and immunogenicity in SIV-infected Rhesus macaques (a model of human HIV-1 infection) are unique features of ML29 ^92^. In West Africa, LF-endemic areas (for example, Nigeria) also have high HIV seroprevalence, and it is important to determine whether vaccination could be safe in the context of HIV-1 infection.

## Do current vaccine candidates meet World Health Organization guidelines?

**Figure 2.**
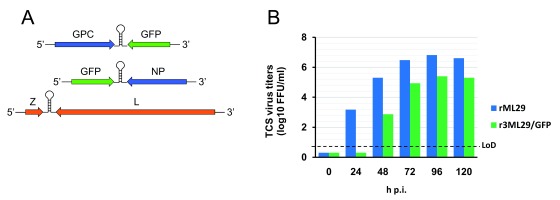
Generation of r3ML29. BHK-21 cells were transfected with pol-II expression plasmids for ML29 L and NP, required to support viral replication and transcription, together with plasmids that direct pol-I-mediated intracellular synthesis of L, and recombinant S1 and S2 RNA genome species. Six days later, tissue culture supernatants were collected and used to infect Vero cells to amplify the rescued r3ML29 that were plaque-purified and scaled up to generate viral stocks. ( **A**) Genome organization of r3ML29/eGFP. ( **B**) r3ML29/EGFP grows to high titers in Vero cells. Cells were infected (multiplicity of infection = 0.1) and virus titers in tissue culture were determined at the indicated times. The rML29 reverse genetics is an advanced vaccine platform to further improve the safety and immunogenicity of Lassa virus vaccine candidates. FFU, focus-forming units; GFP, green fluorescent protein; GPC, glycoprotein precursor; h.p.i., hours post-infection; LoD, limit of detection; NP, nucleoprotein; TCS, tissue culture supernatant.

In 2017, the WHO released the Target Product Profile (TPP) for an LASV vaccine ^94^ and emphasized the preventive use of the vaccine as the highest priority. Optimal candidate vaccines should fulfill the following criteria: (i) WHO-acceptable safety/reactogenicity, (ii) an injectable single-dose regimen, (iii) high (≥70%) efficacy in preventing infection or disease caused by LASV lineages I to IV, and (iv) confers long-lasting (≥5 years’) protection. Several virus-vectored vaccine candidates based on vaccinia virus ^50, 74, 95^, recombinant vesicular stomatitis virus (rVSV) ^96, 97^, MOPV ^98, 99^, ML29 ^86, 87^, yellow fever 17D ^100, 101^, and alphavirus replicons ^102, 103^ have been tested in proof-of-concept studies in NHPs ( Table 1). Among them, ML29 and rVSV/LASV-GPC have been recommended for accelerated vaccine development by international vaccine experts ^104^. ML29 exhibits unique features to meet WHO TPP criteria: (i) safety in all available animal models of LF, including immunocompromised NHPs ^92^; (ii) induction, after a single shot, of protective sterilizing T-cell responses against SL and Nigerian strains of LASV ^46, 105^ responsible for the ongoing outbreak in Nigeria ^68^; (iii) genetic stability *in vivo* and *in vitro* ^106^; (iv) efficacy in post-exposure applications ^107^; and (v) favorable thermostability. The low dose of ML29 (1 × 10 ^3^ plaque-forming units) required for the induction of protective immunity makes this vaccine attractive for manufacturers. This dose is in the range of the natural infection dose during rodent-to-human transmission ^7^ and presents LASV NP- and CPC-derived epitopes to the major histocompatibility complex (MHC) molecules in the most effective way to induce robust cross-protective T-cell responses ^46, 87, 88, 105^. Depletion of CD8 ^+^ T cells in ML29-immunized animals completely abolished protection, whereas CD4 ^+^ T cells had partial effect ^108^. A recombinant ML29 (rML29) has recently been rescued from cDNA clones ( Figure 2). Arenavirus tri-segmented (r3) technology ^111, 112^ would allow for the generation of r3ML29 expressing additional LASV antigens to expand the cross-protection range of ML29-based LASV vaccines (for example, expressing GPC and NP antigens from distantly related lineage I, LASV/LP).

**Table 1.** Advanced LASV vaccine candidates tested in “proof-of-concept” efficacy trials in NHPs.

Vaccine candidate	LASV vaccine antigen formulation	Vaccine regimen	Efficacy against LASV/JOS ^a^	Efficacy against LASV/NIG ^b^	Viremia after challenge ^c^	Correlates of protection	Ref
Recombinant vaccinia virus	GPC (JOS) NP GPC&NP	Single vaccination, at four sites, total 1x10 ^9^ PFU, ID	88% 20% 100%	ND	Low–moderate High ~LD	CMI	^50, 74, 95, 109^
Reassortant MOPV/LASV, ML29	GPC&NP (JOS)	One dose, 1x10 ^3^ PFU, SC	100%	100%	<LD	Sterilizing CMI	^46, 87, 92, 105– 107^
rVSVΔG/LVGPC	GPC (JOS)	One dose, 1–6x10 ^7^ PFU, IM	100%	ND	Low, transient	nAbs? CMI?	^48, 97^
YF17D/LASV	GPC (JOS)	Two doses, 1x10 ^7^ FFU, SC	20%	ND	Moderate–high	ND	^100, 101^, Lukashevich, unpublished
VEEV-TC83 RNA replicon particles	GPC (JOS&LP) ^d^	Two doses, 1x10 ^7^, SC	80%	20%	Moderate	ND	^102, 103^, Lukashevich, unpublished
MOPEVAC _LASV_	GPC (JOS)	One dose, 6x10 ^6^ PFU/dose, SC	100%	ND	ND	nAbs, CMI	^99^
DNA	GPC (JOS)	Two immunizations, 20 mg DNA at four sites, ID electroporation	100%	ND	ND	nAbs?	^110^

^a^ Challenge dose: 1x10 ^3^–1x10 ^4^ PFU of LASV/JOS (lineage IV), route of inoculation: SC or IM. LASV-Z32 (Liberia, lineage IV) was also used in vaccination/challenge experiments with rVSVΔG/LVGPC ^47^.

^b^ Nigerian strain of 803213 (lineage II) was used in vaccination/challenge experiments with ML29 vaccine in marmosets. This strain causes fatal disease mimicking LF human hepatitis and features of arenaviral hepatitis in a murine model ( Figure 1).

^c^ Low–moderate, 10 ^3^–10 ^4^ PFU/mL; high, >10 ^4^ PFU/mL.

^d^ The genetic backbone of VEEV TC-83 vaccine was used to design bicistronic RNA replicons encoding wild-type LASV-GPC (JOS or LP) and C-terminally deleted, non-cleavable modified glycoproteins fused with fibritin. Bicistronic replicons were encapsidated into virus-like-particles using VEEV capsid and glycoproteins provided in *trans* ^94^.

Abbreviations: CMI, cell-mediated immunity; FFU, fluorescent forming units; GPC, glycoprotein; GPC&NP, simultaneous expression of NP and GPC in the same vector; ID, intradermal; IM, intramuscular; LASV, Lassa virus; LD, limit of detection; nAbs, neutralizing antibody responses; ND, not done; NHP, non-human primate; NP, nucleoprotein; PFU, plaque-forming unit; SC, subcutaneous.

Reverse genetics has also been used to generate more attenuated MOPV by disrupting the 3'–5' exonuclease (Exo) domain of NP required for its anti-IFN activity ^99^. LASV/JOS GPC gene was cloned and expressed in MOPVAC _ExoN6b_, and a single immunization of MOPEVAC _LASV_ fully protected NHPs from a homologous lethal challenge. However, disrupting NP Exo resulted in about 100-fold yield reduction of MOPEVAC _LASV_ production in cultured cells ^99^, which combined with a required high dose (6 × 10 ^6^ focus-forming units) will pose obstacles for manufacturing and developing this vaccine candidate.

A single injection of the rVSV/LASV-GPC experimental vaccine where LASV-GPC was substituted for VSV-G fully protected guinea pigs and NHPs against LASV strains from the same lineage ^48, 97^. The rVSVΔG/LASV-GPC immunization did not induce detectable levels of T-cell responses before challenge, and low humoral responses in IgG enzyme-linked immunosorbent assay were found in three out of four vaccinated animals. After LASV challenge, neutralizing antibodies were detected in all four macaques ^97^.

The Coalition for Epidemic Preparedness Innovations (CEPI) ( http://cepi.net) was recently created as a non-profit organization to accelerate vaccine development against emerging epidemic infections when the commercial market is insufficient to justify private investment ^113^. CEPI supports the development of vaccine candidates from the late pre-clinical stage to phase II and stockpiling for emergency use. The CEPI initiative has been widely welcomed and supported by multiple governments, philanthropic organizations, and industrial partners. The development of LASV vaccines was included in an aggressive CEPI Business Plan (2017–2021) with the goal of testing safety and efficacy in human phase IIa/b trials and stockpiling. One of the first LASV vaccine candidates supported by CEPI is being developed by Themis Bioscience and is based on recombinant measles vaccine technology from Institute Pasteur ( www.themisbio.com). The measles virus vector-based vaccine platform looks promising for human infections with antibody-based protection ^114^. However, there are no peer-reviewed pre-clinical data to support the application of this platform for the development of an LF vaccine with a predominant T-cell-mediated mechanism of protection. Pre-existing anti-vector immunity, as it might affect the outcome of clinical studies, has to be addressed for this approach. CEPI is also supporting a DNA-based vaccine against LASV ^115^, which raises feasibility questions. There is no DNA preventive vaccine licensed for human use. Scientific interest on DNA vaccine endeavors has decreased because of difficulties in improving DNA immunogenicity ^116^. Further advancement of plasmid formulations and delivery will be required to see the first licensed DNA vaccine for people “in the next 20 years” ^117^. LASV infection is not an easy target for DNA vaccination since both antigens—NP and GPC—have to be included in the vaccine formulation ^118^. Electroporation of LASV-GPC DNA induced modest levels of neutralizing antibodies, providing additional evidence that T-cell responses played a major role in the protection of NHPs ^110^.

Two additional CEPI awards were announced to support rVSV-based LASV vaccines. The rVSVΔG/LASV-GPC is based on the same platform applied to design the rVSVΔG/ZEBOV-GP vaccine, which was tested in a ring vaccination trial in Guinea ^119, 120^ and which appeared to provide some protection, but its efficacy remains to be determined ^121^. Clinical studies documented side effects associated with the rVSVΔG-based vaccine, including post-vaccination arthritis, detection of vector RNA in blood, and infectious vaccine virus in the skin of vaccinated individuals ^122, 123^. Animal studies were not always in line with Ebola virus (EBOV) vaccine clinical trials, suggesting that immune correlates of protection are not universal and depend on vaccine platform, vaccine regimen, host genetics, pre-existing immunity, immunization schedule, and challenge protocol ^124^. This lesson from EBOV vaccine development is applicable to LASV, since dependence on vaccine platform and vaccine recipients has been documented ^48, 50, 74, 102, 109^ ( Table 1). The second rVSV-based LASV vaccine candidate supported by CEPI is based on the VesiculoVax platform licensed by Profectus BioSciences. This platform was developed to further improve the safety of the rVSV vector by N gene translocation and truncation of VSV-G cytoplasmic tail (CT1). The attenuated rVSV-N4CT1 vector was immunogenic in NHPs and induced only a mild inflammatory response after intra-thalamic inoculation ^125^. This vector was used to generate experimental vaccines expressing single or multiple GPs that protected NHPs against EBOV and MARV challenges ^126– 128^, but there are no peer-reviewed pre-clinical data in support of the LASV vaccine based on the VesiculoVax platform. Likewise, CEPI supported pre-clinical development of the LASV-GPC-based vaccine vectored by non-replicating simian adenovirus ChAdOx1 ^129^, but there is no evidence supporting the suitability of this platform to control LF on the basis of natural history of the disease and mechanisms of protective immunity ^54, 74, 77, 130^.

## Conclusions

Protection against Nigerian strains of LASV is a critical requirement for LASV vaccines ^94^. WHO pre-qualification requirements raise a high bar for vaccine developers and emphasize the WHO-preferred vaccine candidate as a preventive and cost-effective measure for the general population in endemic areas. Among currently available LASV vaccine candidates, only ML29 has been shown to protect against LASV strains from lineage II, which is responsible for the current LF outbreak in Nigeria ^46, 68, 88^.

The “stockpiling” for emergency use is a workable concept for infections with unknown epidemiology and unpredictable outbreaks (for example, EBOV, Nipah, and severe acute respiratory syndrome) but poorly justifiable in the case of LASV, for which endemic areas are well defined and natural reservoir and key routes of human transmission are known. Population-based vaccination is the most effective option to control LF in endemic areas. The successful story of Candid #1, a live-attenuated vaccine against Argentine hemorrhagic fever ^131^, provides a guideline for LASV vaccine clinical development. Because of the high LASV genetic diversity, multicenter trials in several endemic areas (countries) would probably be required to evaluate the cross-protective efficacy of vaccine candidates. Pre-existing LASV immunity potentially affects vaccine dose-dependent responses, and appropriate efforts must be applied during human trial design to correctly assess vaccine immunogenicity. Rooted research collaborations and capacity building will be crucial for anticipating multicenter immunogenicity and efficacy trials in West Africa ^132, 133^.

The WHO-preferred LASV vaccine should cover both adult and pediatric populations. It seems reasonable to plan pediatric studies after the successful completion of phase II trials in adults. Pediatric studies are subject to special provisions of the clinical trial regulations. However, it should be noted that replication-competent US Food and Drug Administration (FDA)-approved pediatric vaccines include reassortant influenza (FluMist, MedImmune) and human-bovine rotavirus (RotaTeq, Merck) vaccines. The safety and efficacy of these vaccines in children and infants indicate that reassortant vaccine platforms are well suited for the pediatric population. The ML29 LASV vaccine can be safe in children as well, since safety and immunogenicity in SIV-infected Rhesus macaques have already been documented ^92^.

The FDA does not recommend replication-competent vaccines for pregnant women, an LF risk group with a high fatality rate, especially in the third trimester of pregnancy. Replication-deficient Modified Vaccinia Ankara vector generating LASV-like particles (MVA-VLP, GeoVax Labs) or inactivated recombinant vaccine candidate expressing LASV-GPC in rabies vaccine vector (LASSARAB) ^134^ can be potentially considered as vaccine candidates for this group of risk. Currently, these vaccines are in very early pre-clinical steps of development.

The CEPI LASV vaccine portfolio is based on different features of vaccine platforms rather than the natural history of the disease and rational vaccine design. The head-to-head comparison of LASV vaccine candidates in validated NHP models under supervision of unbiased internationally recognized experts to assess cross-protective breadth seems a very reasonable step before going into expensive clinical phase II trials ^93^. This comparison, combined with phase I safety data, will provide very valuable information for further clinical development to target different groups at risk in endemic areas of West Africa.
